# Estimates of seasonal influenza‐associated mortality in Bangladesh, 2010‐2012

**DOI:** 10.1111/irv.12490

**Published:** 2017-12-02

**Authors:** Makhdum Ahmed, Mohammad Abdul Aleem, Katherine Roguski, Jaynal Abedin, Ariful Islam, Kazi Faisal Alam, Emily S. Gurley, Mahmudur Rahman, Eduardo Azziz‐Baumgartner, Nusrat Homaira, Katharine Sturm‐Ramirez, A. Danielle Iuliano

**Affiliations:** ^1^ International Centre for Diarrheal Disease Research, Bangladesh (icddr,b) Dhaka Bangladesh USA; ^2^ The University of Texas Health Science Center at Houston Houston TX USA; ^3^ The University of Texas MD Anderson Cancer Center Houston TX USA; ^4^ Centers for Disease Control and Prevention Atlanta GA USA; ^5^ Institute of Epidemiology, Disease Control and Research (IEDCR) Dhaka Bangladesh; ^6^ School of Women's and Children's Health The University of New South Wales (UNSW) Sydney NSW Australia

**Keywords:** Bangladesh, burden, influenza, mortality, respiratory, seasonal

## Abstract

**Background:**

Seasonal influenza‐associated mortality estimates help identify the burden of disease and assess the value of public health interventions such as annual influenza immunization. Vital registration is limited in Bangladesh making it difficult to estimate seasonal influenza mortality.

**Objectives:**

Our study aimed to estimate seasonal influenza‐associated mortality rates for 2010‐2012 in Bangladesh.

**Methods:**

We conducted surveillance among hospitalized patients with severe acute respiratory illness (SARI) for persons aged ≥5 years and severe pneumonia for children <5 years in 11 sites across Bangladesh. We defined the catchment areas of these sites and conducted a community survey in 22 randomly selected unions (administrative units) within the catchment areas to identify respiratory deaths. We multiplied the proportion of influenza‐positive patients at our surveillance sites by the age‐specific number of respiratory deaths identified to estimate seasonal influenza‐associated mortality.

**Results:**

Among 4221 surveillance case‐patients, 553 (13%) were positive for influenza viruses. Concurrently, we identified 1191 persons who died within 2 weeks of developing an acute respiratory illness within the catchment areas of the surveillance hospitals. In 2010‐2011, the estimated influenza‐associated mortality rate was 6 (95% CI 4‐9) per 100 000 for children <5 years and 41 (95% CI 35‐47) per 100 000 for persons >60 years. During 2011‐2012, the estimated influenza‐associated mortality rate was 13 (95% CI 10‐16) per 100 000 among children <5 years and 88 (95% CI 79‐98) per 100 000 among persons aged >60 years.

**Conclusions:**

We identified a substantial burden of influenza‐associated deaths in Bangladesh suggesting that the introduction of prevention and control measures including seasonal vaccination should be considered by local public health decision‐makers.

## 1 INTRODUCTION

Estimating influenza‐associated mortality can help assess the burden of influenza disease, identify high‐risk groups, and assist policy makers in determining the value of influenza prevention and control measures. In industrialized countries, excess influenza‐associated respiratory and circulatory deaths are often estimated with regression models using robust vital statistics and viral surveillance time‐series.^1^, ^2^ However, this modeling approach is not possible in many low‐resource settings, such as Bangladesh, because of the lack of reliable vital registration data. In Bangladesh, a national population‐based system to systematically count or categorize deaths using the international classification of diseases (ICD) codes is lacking. While there is an in‐patient death registry system, the population coverage of the system is not comprehensive or nationally representative and a substantial proportion of deaths take place outside the healthcare system, especially in rural areas indicating that many deaths are not captured by this system.^3^, ^4^ Therefore, regression models may not be an appropriate method for estimating influenza‐associated mortality in Bangladesh. However, Homaira et al^5^ adapted a method used for estimating the burden of Japanese encephalitis in Bangladesh^6^ to estimate mortality associated with influenza during the 2009 pandemic. They used sentinel influenza surveillance in four sites and a community‐based death survey to estimate an influenza‐associated mortality rate of 11 per 100 000 in 2009. Since the study was restricted to the 2009 pandemic year, and surveillance was conducted only two days per month, they had limited numbers of influenza‐positive samples (60 specimens overall and only five from children <5 years) to generate these estimates.

Estimates using data from non‐pandemic years and from more geographic locations throughout the country would better assess the burden of seasonal influenza in Bangladesh to inform possible prevention and control measures. Further, burden from seasonal influenza epidemics has been shown to differ from the burden associated with the emergence of a pandemic virus.^7^ The aim of this study was to estimate seasonal influenza‐associated mortality rates across Bangladesh using sentinel surveillance from multiple sites representative of all divisions of the country and community survey data collected during 2010‐2012.

## 2 METHODS

**Figure 1:**
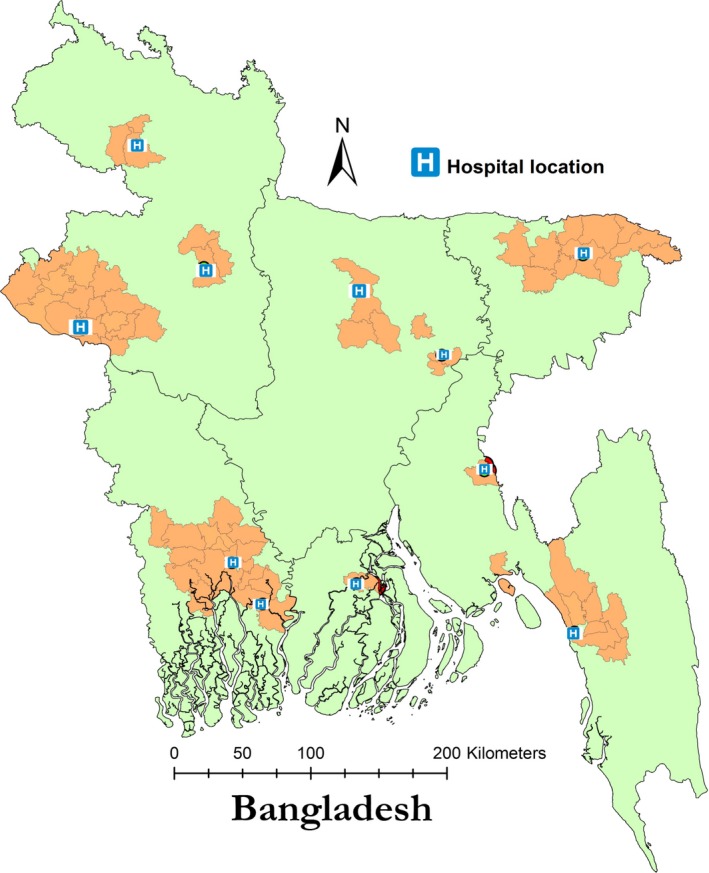
Locations of 11 surveillance hospitals and the catchment areas of these hospitals, Bangladesh

We expanded study sites and revised analytical methods from the previous study^5^ to estimate seasonal influenza‐associated deaths. Virology data were collected from influenza sentinel surveillance hospitals during 2010‐2012 (Figure 1). Twelve hospitals (six governments and six private) located throughout Bangladesh and representing all administrative divisions of the country were selected as sentinel sites using selection criteria described previously.^8^ Respiratory deaths were identified through community surveys conducted in the populations served by the sentinel hospitals to identify individuals who died within 2 weeks of developing acute respiratory illness (ARI) during the same time period. Age‐specific monthly estimates were then used to calculate the number of influenza‐associated deaths.

### 2.1 Hospital‐based influenza surveillance (HBIS) system

The hospital‐based influenza surveillance (HBIS) system includes a network of 12 tertiary facilities across all eight divisions of Bangladesh.^8^ Eleven sites were included to estimate influenza mortality; it was not practical to define the catchment for the twelfth surveillance site in the capital city of Dhaka because individuals may travel from all over the country to seek care in Dhaka (Figure 1). We included one site representative of the Dhaka division in our study, although the metropolitan capital city of Dhaka was not included. Physicians in each surveillance hospital collected nasal swabs from all hospitalized case‐patients meeting the WHO standardized case definition for severe pneumonia or severe acute respiratory illness (SARI) on a daily basis. Severe pneumonia case‐patients were defined as children aged <5 years who had cough or difficulty breathing with one of the following: lethargy or unconsciousness, convulsions, vomiting and inability to drink, or breastfeed; SARI case‐patients were defined as hospitalized patients aged ≥5 years who had fever and cough or sore throat.^9^ Specimens collected from case‐patients were refrigerated and then transferred daily to a nitrogen dewar until shipment to the laboratory at the International Centre for Diarrheal Disease Research, Bangladesh (icddr,b) in Dhaka, every fortnight. Nasal swabs were tested for influenza A and B viruses by real‐time reverse transcription polymerase chain reaction (rt‐rPCR). Influenza A viruses were further subtyped for seasonal A(H1N1), A(H1N1)pdm09, and A(H3N2) as previously described.^10^

We obtained in‐patient surveillance data and laboratory specimens for 24 consecutive months (May 2010 to April 2012). Hospitalized patients were recruited for participation through a protocol approved by the institutional review board (IRB) at icddr,b. All the samples were collected after taking written informed consent from the patients.

### 2.2 Defining the catchment areas of 11 HBIS hospitals

To identify communities served by the hospitals that participated in HBIS, we first reviewed the hospital registration logbooks for each sentinel site and collected residence information for each admitted patient. We defined the hospital catchment area as subdistricts where most (ie., ≥75%) of the enrolled SARI case‐patients resided^11^ (Figure 1). We obtained data on population size for the communities living in these catchment areas from the 2011 Bangladesh Bureau of Statistics national census.^12^

### 2.3 Community survey to identify deaths

We conducted a community survey in randomly selected unions in each hospital catchment area to identify persons who had died within 2 weeks of developing ARI. A union is the lowest administrative subdivision in Bangladesh, analogous to a county in the United States. We defined ARI‐associated deaths in the community as follows: for children <5 years, sudden onset cough or difficulty in breathing within 2 weeks of death and for persons ≥5 years, sudden onset fever and cough or sore throat within 2 weeks of death. For both age‐groups, the disease had to be a new onset and not a progression of a known pre‐existing condition such as tuberculosis. We randomly generated two global positioning system (GPS) coordinates within each catchment area of the 11 sentinel surveillance sites. We conducted the community survey in the unions where these 22 GPS coordinates were located. During July to December 2012, our field team visited approximately 200 villages in these unions. We first interviewed people at key social gathering points, such as community centers, mosques, village courtyards, tea‐stalls, and village markets. We asked individuals if they were aware of any deaths in the area from May 2010‐April 2012.

We also consulted village doctors and leaders of local mosques to gather information about deaths in that particular area during the same time period. Based upon the information gathered from the community, we then compiled a list of neighborhoods where at least one person died during the study period.^6^ Our field team then visited each decedent's household and queried members who had cared for them about their illness episode preceding death. Multiple proxy respondents were interviewed. A standardized questionnaire was used to record demographics, symptoms, and healthcare seeking during the illness. The team did not complete the survey if the death was attributed to injury, drowning, homicide, or other physical trauma by household members or informants. The field team had prior experience conducting such surveys.^5^, ^6^

We obtained written informed consent from the heads of households before conducting the survey at the decedent's house. The protocol was approved by the IRB at icddr,b, and CDC reliance on a non‐CDC IRB was obtained.

### 2.4 Classifying deaths

We classified respiratory deaths in the unions as being associated with severe pneumonia and SARI, according to the case definitions used by HBIS (described above) matching the age‐groups. However, in HBIS, physicians recorded illness signs/symptoms, whereas in the community survey, illness information was recorded retrospectively by our field team from the narratives of caregivers. A death was classified as being potentially associated with an ARI if the patient met the case definition for severe pneumonia or SARI and died within 14 days of onset during the same illness episode. The standard SARI case definition requires hospitalization but for the community reported SARI, we considered the symptoms‐only and hospitalization was not a requirement.

### 2.5 Data analysis

We calculated influenza‐associated mortality rates for the following age‐groups: 0‐4 years, 5‐60 years, and >60 years. To calculate, we first computed monthly mortality rates by multiplying the proportion of severe pneumonia and SARI case‐patients from all surveillance hospitals that tested positive for influenza for all ages by the number of respiratory deaths in the community in a specific month and age‐group. Then, we divided this numerator by the age‐specific census 2011 population of the 22 unions.^12^ As influenza proportion‐positive may vary between age‐groups and there may be bias in who seeks care and is enrolled in surveillance, we only used influenza proportion‐positive for all ages in our calculation of mortality rates for each month. We added monthly estimates for a consecutive 12 month period to calculate the annual mortality rate for 2010‐2011 and 2011‐2012. The analytical methods were adapted from the WHO manual for estimating disease burden for seasonal influenza.^11^ We used the following equation to calculate mortality rate:

$$I_{a,y}=\frac{\sum_{m=1}^{12}D_{a,m,y}\times F_{m,y}\times 100,000}{P_{a,y}}$$

where,

*I*_*a,y*_=rates of influenza mortality per 100 000 persons by age‐group (a) and year (y).

*D*_*a,m,y*_=number of ARI‐deaths by age‐group (a), month (m), and year (y).

*F*_*m,y*_=proportion of influenza virus positive in surveillance hospitals, by month (m) and year (y).

*P*_*a,y*_=census population by age‐group (a) and year (y).

To calculate 95% confidence intervals (CI) for the mortality estimates, we calculated the variance of the influenza virus proportion‐positive for each month of our study period assuming a negative binomial distribution and the variance around ARI‐associated deaths assuming a Poisson distribution. We computed the variance of influenza‐attributed ARI‐deaths for each month using the following equation:

Variance=(N*F)+(N*F ^*2*^)+(F*N ^*2*^).

where,

*N*=the number of respiratory deaths in the community.

*F*=variance of influenza positivity.

We used the following formula to generate the 95% CI for the incidence rates:

95% CI=*I*_*a*_ ±1.96 *(√σ).

where,

*I*_*a,y*_=rates of influenza mortality per 100 000 persons by age‐group (a) and year (y).

σ=variance of the *I*_a_ (sum of monthly variances for 12 consecutive months).

To calculate a national estimate of influenza‐associated deaths, we applied the age‐specific influenza‐associated mortality rates calculated using the formulas described above to the 2011 census population estimates for Bangladesh. We applied the rate from sentinel sites including its variance to the total population of Bangladesh.

## 3 RESULTS

### 3.1 Hospital‐based surveillance

**Figure 2:**
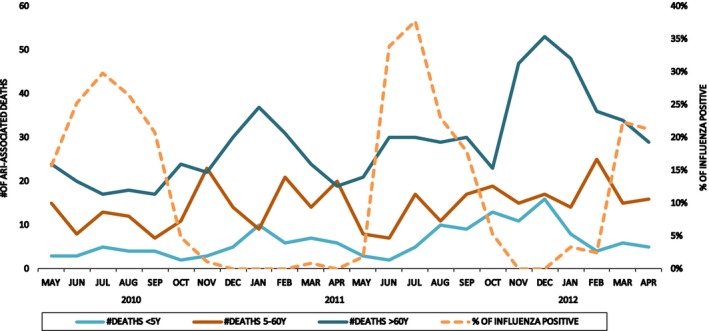
Number of acute respiratory illness (ARI)‐associated deaths among each age‐group, by month and overall proportion of influenza virus‐positive patients per month across all sentinel hospitals from May 2010‐April 2012, Bangladesh

During our study period, physicians collected nasal swabs from 4221 case‐patients (SARI and severe pneumonia), of which 553 (13%) were positive for influenza viruses. Across 11 study sites, a median of 399 (IQR 351‐423) samples was tested for influenza and a median of 46 (IQR 35‐63) samples was positive for influenza per site. Of the 4221 samples, 1911 (45%) were collected during peak period of influenza activity. Among the 553 influenza‐positive patients, 439 (79%) were collected during peak influenza period, 498 (90%) were SARI cases, and 55 (10%) were severe pneumonia cases. The average annual percent positive was 4% (95% CI: 2‐5%) among children <5 years and 18% (95% CI: 13%‐26%) for people aged ≥5 years. The influenza percent positive ranged from 0% in December 2010 to 40% in July 2011 across all ages (Figure 2). Among the 553 influenza‐positive samples, 233 (42%) were collected during 2010‐2011 and 320 (58%) were collected during 2011‐2012. In 2010‐2011, 39% of influenza‐positive samples were A(H1N1)pdm09, 37% were influenza B, and 24% were A(H3N2). In 2011‐2012, 43% of influenza‐positive samples were A(H3N2), 29% were influenza B, and 28% were A(H1N1)pdm09. The influenza subtype distribution was similar between the study sites.

#### 3.1.1 Catchment areas and community survey

The 11 HBIS sites had a total catchment area of 370 unions and the population living in these catchment areas represented about 15% of the population of Bangladesh.^12^ The 22 randomly sampled unions from the catchment had a total population of about 737 165, with an average population of 33 500 per union.

We identified 10 092 total deaths in the 22 surveyed unions, of which 1191 (12%) deaths were classified as ARI‐associated deaths and 453 (38%) of those were females. The median age of deceased children with reported ARI was 3 months (interquartile range [IQR] 1‐11 months), and the median age of adults was 70 years (IQR 50‐75 years). Among the ARI‐associated deaths, 150 (12.5%) were among children aged <5 years of age and 693 (58%) were among persons aged >60 years (Table 1). Of the 1191 ARI‐associated decedents, 616 (52%) received treatment by a certified physician as an outpatient and 449 (38%) were hospitalized during the illness episode preceding death.

**Table 1:** Characteristics of acute respiratory illness (ARI)‐associated deaths (N = 1191) in 22 unions in Bangladesh during 2010‐2012

Characteristics	<5 y n (%)	5‐60 y n (%)	>60 y n (%)	All ages n (%)
aTotal ARI‐associated deaths	150	348	693	1191
Female	70 (47)	140 (40)	243 (35)	453 (38)
Smoker	0 (0)	171 (49)	432 (62)	603 (51)
bSought treatment from				
Certified doctor (outpatient)	87 (58)	202 (58)	327 (47)	616 (52)
cUncertified provider	35 (23)	81 (23)	256 (37)	372 (31)
Pharmacy/drug seller	2 (1)	12 (3)	17 (2)	31 (3)
Self/other	15 (10)	53 (15)	93 (13)	172 (14)
Hospitalized	82 (55)	169 (48.5)	198 (28.5)	449 (38)
Female	37 (25)	62 (18)	56 (8)	155 (13)
Died at home	57 (38)	170 (49)	485 (70)	712 (60)
Female	30 (20)	71 (20)	184 (26.5)	285 (24)

a Identified as having died within 14 days of ARI.

b Treatment for the episode of ARI preceding death.

c Traditional healers with no formal certification.

Seven hundred and twelve (60%) of the ARI‐associated deaths occurred at home. Among the 693 decedents who were aged >60 years, 614 (89%) had pre‐existing health conditions. Of the comorbid conditions, respiratory diseases (33%), cardiac diseases (21%), and cancer (24%) were most commonly reported. We observed higher ARI‐deaths (N = 727/1,191) in the months of October‐April (Figure 2), which are colder months in Bangladesh.^8^ The percent of samples testing positive for influenza was at its lowest (0%‐2%) during the same time period (Figure 2). We also observed 464 ARI‐associated deaths during the months of May to September when the average monthly influenza‐positive proportion was 15% in 2010‐2011 and 21% in 2011‐2012.

#### 3.1.2 Influenza‐associated mortality estimates

We estimated that the influenza‐associated mortality rate among all age‐groups was six (95% CI: 3‐14) per 100 000 population in 2010‐2011 and 11 (95% CI: 2‐25) in 2011‐2012 (Table 2). Rates of influenza‐associated death were highest in persons aged >60 years (41 [95% CI: 35‐47] in 2010‐2011; 88 [95% CI 79‐98] in 2011‐2012) (Table 2). After extrapolating the influenza‐associated mortality rates to the 2011 census population of Bangladesh (ie., 144 043 697)^12^, we estimated 6097 (95% CI 2604‐14 199) persons died across all ages in 2010‐2011 and 16 804 (95% CI: 8588‐25 019) died during 2011‐2012. Approximately, 80% of the influenza‐associated deaths in 2010‐2011 (ie., 4732 [95% CI: 3067‐5396]) occurred among adults aged >60 years and about 60% (ie., 10 207 [95% CI 9114‐11 300]) in 2011‐2012.

**Table 2:** Estimated influenza‐associated mortality rate per 100 000 population in the catchment areas of 11 tertiary hospitals in Bangladesh, 2010‐2012

Age‐groups	Number of ARI‐associated deaths	aAverage annual influenza proportion‐positive (95% CI)	Population of b22 unions	Influenza‐associated mortality per 100 000 in 2010‐2011 (95% CI)	Influenza‐associated mortality per 100 000 in 2011‐2012 (95% CI)
All ages	1191	13% (12‐14)	737 165	6 (2‐14)	11 (2‐ 25)
<5 y	150	4% (2‐5)	75 782	6 (4‐9)	13 (10‐ 16)
5‐60 y	348	20.5% (17‐23)	603 442	2 (1‐6)	4 (2‐ 9)
>60 y	693	16% (13‐19)	57 960	41 (35‐47)	88 (79‐ 98)

ARI, acute respiratory illness.

a The average annual age‐specific influenza proportion‐positive was not used in the incidence calculation; monthly influenza virus percent positive across all ages was used.

b The 22 unions were randomly selected from 370 unions in the hospital catchment areas.

## 4 DISCUSSION

In 2010‐2011 and 2011‐2012, we estimated between six and 11 influenza‐associated deaths per 100 000 populations at 11 sentinel sites for all age‐groups. We estimated the highest burden among persons aged >60 years for both periods. Our data came from a nationally representative sample spanning all administrative divisions of Bangladesh, suggesting that these mortality rates may be representative of the burden of seasonal influenza across the country. Our observed mortality rate for 2011‐2012 was similar to the 2009 estimate. However, in contrast with the same study,^5^ we conducted influenza surveillance each day of the week across a greater number of sites during 2010‐2012. We also observed a higher proportion of A(H1N1)pdm09 circulating, and therefore, more deaths were attributed to A(H1N1)pdm09 in the current study. While the previous study was important for understanding deaths due to influenza occurring in 2009, the current study identified burden during two consecutive seasonal epidemic periods. The observed influenza burden suggests the possible value of exploring the cost‐benefit of influenza prevention and control measures, especially among young children and older adults.

Our observed mortality rate is consistent with data from other countries. In China, the rate of influenza‐associated mortality attributed to respiratory and circulatory disease was estimated to be 12.4 per 100 000 annually for three northern cities and 8.8 per 100 000 annually in five southern cities.^13^ One difference in methods was circulatory deaths were included in the Chinese estimates, which our methods were not designed to capture.

In Thailand, the average annual influenza‐associated mortality rate was estimated as 4.0 per 100 000 persons, which is lower than our estimates, although there is considerable overlap of the confidence intervals.^14^ In Thailand, the average influenza‐associated death rate among ≥65 years was 42 per 100 000 annually, which is similar to our estimate for 2010‐2011 of 41 (95% CI 35‐ 47) among those aged >60 years.

In South Africa, the incidence of influenza‐associated SARI deaths was estimated to be 20.1/100 000 annually for children <1 year and 10.4 (95% CI 8.4‐12.9) for those aged 45‐64 years.^15^ While our age‐groups were not directly comparable to South Africa, there appears to be lower burden of influenza mortality among children and higher burden among elderly in Bangladesh. Human immunodeficiency virus (HIV) is the leading cause of childhood mortality in South Africa^16^ and the higher influenza‐associated death estimate could potentially be attributed to HIV in South Africa, which is not prevalent in Bangladesh.^17^

In Hong Kong, seasonal influenza mortality was estimated to be 11.1 (95% CI 7.2‐ 14.6) per 100 000 persons annually, which is similar to our estimates.^18^ The government of Hong Kong adopted a policy of free seasonal influenza vaccines for priority groups including the elderly and people living with disabilities.^19^ For 35 countries in the Americas, the estimated annual mean influenza‐associated mortality rate was 31.9 per 100 000 persons among those aged 65‐74 years, which was lower than our estimate for persons aged >60 years in Bangladesh.^20^ Better access to care in this region may result in a lower burden of seasonal influenza compared to Bangladesh.

Previously, May‐September (corresponding to the rainy season) was reported as peak months of influenza virus circulation in Bangladesh^8^. In our study, we also observed higher influenza virus positivity in this period and identified 464 ARI‐associated deaths in the community coinciding with this period. It is also common for influenza viruses to circulate outside the typical season and cause illness^21^, ^22^. We identified 727 ARI‐associated deaths during October to March, which are not peak months of influenza circulation in Bangladesh as observed by our surveillance. This observation of dissociation of peaks of influenza circulation and the peaks of mortality in the community was also reported in Thailand^14^ and after the 2009 pandemic in Hong Kong^18^ when A(H3N2)/A(H1N1)pdm09 peak activity did not correspond with the mortality. These deaths may be due to influenza or other respiratory pathogens, which may also circulate in Bangladesh.^23^

There are some limitations to acknowledge. Our mortality estimates are likely underestimates of true burden of influenza mortality. The survey may have missed deaths, especially among infants who may have presented with sepsis rather than respiratory symptoms.^24^ Even among adults, it is possible that they had an underlying illness that made it difficult to classify as ARI through a community survey. When deaths were identified, it is possible that poor recall may have impacted our ability to identify ARI symptoms among decedents because we asked individuals to remember events that may have occurred more than 1 year before. Nevertheless, we relied on multiple proxy respondents and as death is a rare and traumatic event, it is possible that respondents may better remember symptoms related to such events. By applying the influenza proportion‐positive from hospitalized patients to community deaths, we assumed hospitalized patients have similar proportion of influenza‐positive as among seriously ill persons in the community. In addition, our study was conducted as a survey in 11 sites in Bangladesh and extrapolated to the entire population. Our method to identify deaths is still not a national system of counting or systematically coding deaths and may under or over‐represents the true number of ARI‐associated deaths. Further, we only identified decedents with an acute respiratory illness and did not explore the contribution of circulatory or other causes of death, which could have been associated with influenza deaths.^25^, ^26^ Finally, we did not include the capital city Dhaka, which is the most densely populated city of Bangladesh because we could not define a catchment area for the hospital in Dhaka. However, one sentinel site in our study represents the Dhaka division (Dhaka city is a part of the Dhaka division). An estimate that includes the capital city would be more representative of the country. Even with these limitations, we think our extrapolations to be reasonable estimates of influenza‐associated death for Bangladesh as evidenced by regional comparisons and comparisons with similar middle‐income countries. To validate the model, this methodology can be employed in a country where regression‐based estimates for influenza mortality are available.

Our estimates highlight the importance of continued surveillance and a need to consider preventive measures that could potentially reduce the burden of influenza virus infection in the population. A comparative review of seasonal influenza‐associated mortality burden in low‐ to high‐income countries showed that seasonal influenza vaccine coverage in most of Africa and Asia is less than 1%.^27^ Bangladesh spends about U.S. $31 per capita for health expenditure, compared with a per capita expenditure of U.S $570 by South Africa;^28^ therefore, affordability of influenza vaccine is an important consideration for Bangladesh. While not comparable to Bangladesh in terms of health expenditure, China adopted influenza immunization through both public and private sources with a mortality estimate similar to our current estimates.^29^

In addition to seasonal vaccinations and antiviral drugs, hand washing has been shown to be effective for influenza prevention in some high‐income countries.^30^, ^31^, ^32^ However, a reactive approach to influenza prevention through hand washing was ineffective in rural Bangladesh^33^ suggesting alternative approaches may be required. Hand washing at recommended times, such as before preparing food, has been effective in reducing childhood diarrhea in Bangladesh.^34^ It is worth exploring if emphasizing regular hand washing at key times would be beneficial in preventing influenza in communities.

## 5 CONCLUSION

We reported substantial burden of seasonal influenza in the population of Bangladesh during 2010‐2011 and 2011‐2012. These findings will help policy makers better explore and prioritize strategies for influenza prevention and control in Bangladesh.
